# Diabetic Retinopathy and Ehrlichia: the Possible Relationship

**Published:** 2012

**Authors:** Charles A. Kallick

**Affiliations:** Rush University Medical Center, Chicago, Illinois, USA.

**Keywords:** Ehrlichia, Diaberic retinopathy, Pathophysiology

## Abstract

In the United States, 20,000 patients each year lose their sight from diabetic retinopathy. The cause has been attributed to a failure of control of glucose levels. Recent studies have challenged this, and have suggested that there is no evidence for a consistent glycemic threshold in various populations relating to the incidence of retinopathy. The Ehrlichia have been recently suggested as having a role in diabetes. The action of this obligate parasitic bacterium which often affects the cells involved in immunity has the potential of affecting various tissues randomly. This includes self-reactive T or B cells which may be erroneously altered or released from the marrow because of infection of marrow precursors by an Ehrlichia. The discovery of a gene obtained by molecular methodology from a leukemia patient, has given us a tool to identify by molecular methods, the presence of this gene and assumed bacterium in the blood of patients with various syndromes that includes diabetes. Because of the inconsistent evidence of a uniform glycemic threshold in retinopathy and the failure of its control, this hypothesis raises the question of something else that might be causing this damage. The suspected bacterium in diabetes may have as a significant side effect of its infection of the immune system, specifically the site of action of damage to the small vessels of the retina which could lead to what is regularly described in retinopathy; further, and that may include damage to other vessels seen in peripheral and coronary arteries. The availability of a molecular test in whole blood specimens from diabetics suggests a survey for the gene of the bacterium described in diabetic patients and matched controls. Such an investigation could lead to other therapies directed against the bacterium's presence in the marrow if discovered, and strategies to eliminate the harmful self-reactive T or B cells, if found in diabetes.

## INTRODUCTION

Diabetic retinopathy is a major cause of blindness in type 1 and 2 diabetes. (1) Damage to the retina in diabetes has long been attributed to failure of control of hyperglycemia and control of the glycemic abnormalities has been presented as the major way to prevent the onset of visual loss in both types of diabetes (2). A recent study raises questions about the orthodox view that glycemic abnormalities cause retinal damage. Wong et al., found inconsistent evidence of a uniform glycemic threshold for prevalent and incipient retinopathy, suggesting a discontinuity between cause and effect (3). This finding raises the question of what else might be causing retinal damage in diabetes. Ehrlichia are little-known and poorly understood obligate parasitic intracellular bacteria. Most published data on these bacteria concentrate on the two known agents, believed to be common in rodents, which have been successfully cultured from the blood of patients suffering from acute Ehrlichiosis, following from infection transmitted by ticks. This group is now being expanded (4).

 The Anaplasma are a closely related group of organisms. For purposes of clarity in this paper, they will be referred to collectively henceforth as the EA. 

 A publication in the Journal of Medical Hypotheses last year by this author introduced the possibility of EA infection of immune system cells, particularly those governing the activity of lymphocytes, and a number of serious disease states of otherwise unknown etiology that might be caused by this condition(5). This author has proposed that another, heretofore unknown Erhlichial agent—tentatively identified by a gene 97% coincident with the major surface protein from Anaplasma phagocytophillium (a member of the EA family)—causes chronic infection in humans, capable of producing many of the features and complications of diabetes, including retinopathy. 

## HYPOTHESES

I hypothesize that in the absence of conclusive proof that glycemic abnormalities cause diabetic retinopathy, another cause -infection with EA- could explain the vascular and neurologic changes in the retina of diabetic patients Malfunction of the human immune system caused by EA could induce changes affecting T or B cells, with a direct cellular effect on the retinal vessels or the possible indirect effect of self-reactive B cells producing antibodies against specific tissue in the retinal vessels.

**Supportive data**

**The function of the bone marrow and relationship to occult infection**

The basic study of the immune system, including the metamorphosis from stem cells to the cells of immunity, is not at present a major area of interest of ophthalmologists. Making the connection hypothesized here requires some knowledge of the construction of lymphocytes, the major agent of cellular and antibody immunity within the bone marrow. 

From the stem cell there is a rapid proliferation of lymphocytic and myelocytic cells of immunity. The active cells are reconstituted by marrow mechanisms using fragments of DNA. This reconstruction is random. Approximately 60% of such cells are directed against body tissues, thus these are self-reactive. Before release from the marrow, several regulatory mechanisms within the marrow normally eliminate the self-reactive cells. (6.)

**One infection that may change the function of the immune system**

EA produce a substance intracellularly, named host transcription protein (HTP). This substance is used by the EA to change its intracellular environment to foster and assist the parasite’s growth, development and reproduction. The untoward effect of HTP may affect the reproductive activity of the rapidly proliferating cells in the marrow. 

The impact of HTP on the intracellular environment of the proliferating marrow cells has been shown in some experiments to affect the nuclear instructions from the parent cell to the daughter cell. These abnormalities may include transcription errors, mutations, and errors of immune cell development (7). Cascading impacts from these transcription errors could result in production of self-reactive antibodies, failure of scheduled apoptosis and/or non-scheduled apoptosis of immune cells, and deficiencies in the development or ratios of particular immune system components. The presence and activity of these self-reactive and malfunctioning immune system cells could cause many and varied human pathologies, including the pathophysiology of diabetic retinopathy.

**Changes in marrow cells and their possible relationship with pathological changes in diabetes, including retinopathy**

Unsuspected impacts from malfunctioning immune system cells could impact the mechanical properties and permeability of the retinal blood vessels, leading to the formation of micro aneurysms, retinal hemorrhages, micro retinal infarcts, and in the nerve fiber layer of the retina, deposit cotton wool patches (7). Other known consequences of diabetes are only connected to the hyperglycemic state with difficulty, and not conclusively proved. These include diabetic nephropathy (Kimmelstiel-Wilson nephropathy) Small vessel disease in the lower extremities and changes in the vascular structure of smaller and larger arterial structures such as the coronary arteries could lead to these other manifestations of the pathology of diabetes(8). 

An interesting correlation is presented by other unexplained problems in the diabetic. The small vessel retinal problems of basement membrane thickening, including micro-aneurysms, and pre-retinal neovascularization , which leads to the eventual loss of vision-- is mirrored by other lesions of diabetes, especially in the diabetic foot. The presence of poor circulation in the lower extremities, with gangrene, repetitive infections leading to amputations and an eventual 30% mortality all occur in the lower extremities often with little evidence of large vessel disease. The hypothesis presented here suggests that the basic cause in both of these manifestations could come about because of self-reactive antibodies or cells, which attack a specific component needed by the small blood vessels for maintenance of competence. This same process could also be at work in the microvasculature of the pancreatic islet cells, which cease to function properly in diabetes.

The hypothesis of a single cause occurring at the molecular level offers a better explanation for diabetes. Scientists have concentrated on the fluctuating glucose level in diabetic but that may not explain the disease states, especially since the glucose level has been effectively challenged as causative of retinopathy.

**Figure 1:**
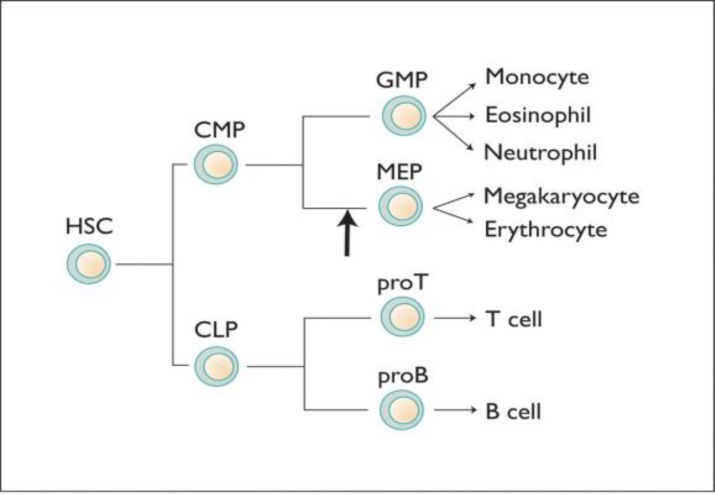
The cascade of the development of the blood cells, including those responsible for immunity, from the stem cell to the active circulating cells of the blood

Human stem cells (HCS) can progress via two major changes to lymphocytic and myelocytic cells. This schematic chart of the functions of the proliferating cells of the bone marrow is indeed remote from the daily considerations of ophthalmologists. In active proliferation of the cells from the bone marrow, which occurs on a daily basis and replaces all of the erythrocytes in the body every 120 days, creates the final cells of immunity especially the T and B cells. These can alter or eliminate the activities of other cells, and the understanding of this basic process is essential when considering the possible activity of Ehrlichia in altering this process (From the Journal of Medical Hypotheses; 77:374-379; 2011).

**Figure 2:**
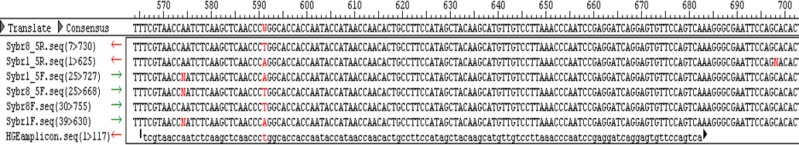
The identification of the sequences described previously from a leukaemia patient’s blood when subjected to PCR which was found to be approximately 97% homologous to the identified major surface protein [MSP2] of *Anaplasma phagocytophillium* [5].

Sybr 8_5R.seq: DNA preparation 8 (5 µl) with the Reverse primer as the sequencing primerSybr 1_5R.seq: DNA preparation 1 (5 µl) with the Reverse primer as the sequencing primer, Sybr 1_5F.seq: DNA preparation 1 (5 µl) with the Forward primer as the sequencing primer, Sybr 8_5F.seq: DNA preparation 8 (5 µl) with the Forward primer as the sequencing primer, Sybr 8 F.seq: DNA preparation 8 (1 µl) with the Forward primer as the sequencing primer, Sybr1F.seq: DNA preparation 1 (1 µl) with the Forward primer as the sequencing primer, HE amplicon.seq: Represents the deposited sequence for Anaplasma phagocytophillium amplified with the Reverse primer, Red T’s and A’s denote the area of divergence of the unknown strain to the positive sequence. This PCR reaction will result in a 70 bp region that can be used for sequencing. In this schematic representation familiar to molecular biologists, explanation is necessary for those not familiar with this highly specialized form of molecular biology. The numbers at the top of the chart represent the position of the standard E Coli used as a reference for bacteria. The extreme bottom part of the chart which is in small non-capitalized letters is the deposited sequences from the BLAST program (HGE is the older nomenclature of human granulocytic Ehrlichia). This has been reported in the BLAST record, and deposited as those found in Anaplasma phagocytophillium (AP). The second line in the chart is the consensuses of those proceeding from the examination of the PCR are from a patient with leukemia. Two aberrations from the sequences of MSP2 of AP occur at numbers 591 and a smaller number at 578. An interpretation of these data suggests that there is indeed a difference from the major surface protein of AP, and the PCR product was obtained from a human patient with myelocytic leukemia. The publication with this data was first presented suggested that the sequences of this result represented a gene found is a pathogen in a human patient with a disease of the immune system specifically, leukemia. The use of the sequences when applied with the proper development of probes and primers could identify the presence or absence of this gene in the whole blood of patients especially diabetics with retinopathy. A statistical analysis of such examination if positive and statistically confirmed gives the ophthalmologists and investigators rationale and tools for intervention to eliminate the presumed invaders of the immune system in the patient. Conclusions derived from these studies could lead to appropriate interventions including antibiotics and other maneuvers to change the immune response which might include marrow transplantation. (From the Journal of Medical Hypotheses; 77:374-379; 2011).

## DISCUSSION

Wong et al. [3] offered a major challenge to the widely accepted view that hyperglycaemia is a definitive factor in diabetic retinopathy. If this group is correct, one must look for another explanation. The hypothesis described in this paper offers alternative mechanisms for inducing these changes at the molecular level, either by antibodies or self-reactive T cells directed against components of the retinal vasculature. 

The mechanism explained in detail here offers a pathologic mechanism by which infection at the stem cell level leads to the unobvious remote changes in the micro-vasculature of the retinal vessels. This hypothesis, if confirmed, could not have been imagined without studying whether or not the infection is present in two groups: diabetic retinopathy or all diabetes. The hypothesis presented here and in the previous paper discussing Ehrlichia and the immune system presents a fertile ground for investigation. Indeed, the bankruptcy of preconceived and believed thought suggested in the Lancet revision should be a spur to investigate all possibilities [3]. 

The sequences of MSP2 that differ from those of AP only at two nucleotides appear to represent the discovery of the gene by molecular methodologies in the whole blood of patients, rather than in controls. These published and in press accounts offer a method of diagnosis to determine whether the suspected bacterium is present or absent. Such a simple study could reveal an alternative explanation for the great malady which afflicts approximately 20,000 diabetics in the United States each year with blindness. If it is found that patients with diabetic retinopathy have the bacteria using the precise diagnosis of molecular methodology, yet controls do not, various studies could and should be devised to see if interventions can alter the course of diabetic retinopathy. Further knowledge must first be obtained to determine whether the defect is in T cells or B cells or both. Interventions will become easier if that information is obtained, and this could be easily discovered. The possible interventions if the presence of the pathogen is confirmed would include antibiotic therapy and/or stem cell replacement with marrow transplants.

## CONCLUSION

It is axiomatic that if an interpretation of data suggests a pathological process, and the suggested treatment of that interpretation of data fails to provide a satisfactory explanation of why that treatment has failed, that a re-examination of the entire process is mandated. This alternative explanation should be considered and tested.

## DISCLOSURE

The authors report no conflicts of interest in this work.
